# Generation of disk-like domains with nanometer scale thickness in merocyanine dye LB film induced by hydrothermal treatment

**DOI:** 10.1186/1556-276X-8-429

**Published:** 2013-10-17

**Authors:** Yasuhiro F Miura, Motoaki Sano, Tsuneyoshi Sugimoto

**Affiliations:** 1 Graduate School of Engineering, Toin University of Yokohama, 1614 Kurogane-cho, Aoba, Yokohama 225-8503, Japan; 2 Department of Clinical Engineering, Toin University of Yokohama, 1614 Kurogane-cho, Aoba, Yokohama 225-8503, Japan

**Keywords:** 68.47.Pe, 68.37.-d, 78.15.-d, Merocyanine dye, Langmuir-Blodgett (LB) film, J-aggregates, Hydrothermal treatment (HTT)

## Abstract

We have characterized the binary LB films of merocyanine dye (MS) and arachidic acid (C_20_) before and after hydrothermal treatment (HTT), which is defined as a heat treatment under relative humidity of 100%, focusing on the morphology studied by bright field (BF) microscopy and fluorescence (FL) microscopy. BF microscopy observation has revealed that the as-deposited MS-C_20_ binary LB film is found to emit intense red fluorescence over the whole film area by 540-nm excitation. Since the surface image is almost featureless, it is considered that the crystallite sizes of J-aggregate are less than 10 μm. Interestingly, after HTT, round-shaped domains are observed in the LB systems, and the sizes are reaching 100 μm in diameter. Crystallites of J-aggregate, which are bluish in color and emit intense red fluorescence, tend to be in the round domains. We have observed two different types of domains, i.e., blue-rimmed domains and white-rimmed domains, which are postulated to be confined in the inner layers and located at the outermost layer, respectively. The thickness of the domains is equal to or less than that of the double layer of the MS-C_20_ mixed LB film, which is *ca.* 5.52 nm. The molecular order of MS in the J-aggregate is improved by the HTT process leading to the significant sharpening of the band shape together with the further red shift of the band (from 590 to 594 nm up to 597 to 599 nm). The reorganized J-band is considered to be ‘apparently’ isotropic owing to the random growth of the J-aggregate in the film plane. We consider that the lubrication effect by the presence of water molecules predominates in the HTT process.

## Background

Recently, J-aggregates formed by organic dyes have been attracting much attention because of their potential application to information storage, energy transfer, and non-linear optical devices. The J-aggregate is characterized by a sharp excitonic band, called J-band, which is remarkably red-shifted from its dye monomer band and an intense fluorescence with zero or small Stokes shift as a consequence of a specific low-dimensional dipole-coupled chromophore array of dye molecules. So far, however, the mechanism of the J-aggregate formation has not been fully elucidated [1].

**Figure 1:**
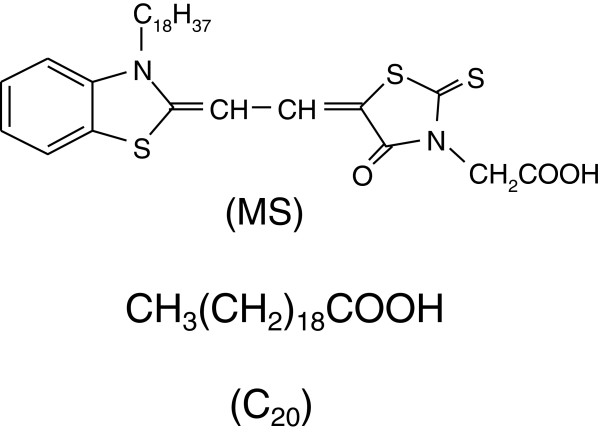
**Molecular structures of merocyanine dye (MS) and arachidic acid (C**_**20**_**).**

The merocyanine derivative with a hydrocarbon chain together with a carboxyl group (MS in Figure 1) has been well known to form J-aggregates in its pure and mixed systems at the air/water interface [2-10]. Since J-aggregates typically consist of dye molecules based on symmetrical chromophores, such as cyanine dyes, the merocyanine dye with both electron donor and acceptor portions in its chromophore is an exceptional and ‘exotic’ constituent for forming J-aggregates [1]. Since merocyanine J-aggregates are not formed under conventional dry conditions, the air/water interface is an exceptional and interesting field for J-aggregate formation, and it is logical to assume that water molecules play key roles for the aggregate formation.

The J-aggregates of MS can be formed on subphases containing divalent metals such as Cd^2+^, Ca^2+^, and Mg^2+^ or on pure water with or without adding matrix molecules [1-12]. Since both of the spectral profile and its stability of the J-band change depending on species of divalent metals and pH, it is assumed that the driving force of the J-aggregate formation is the generation of intermolecular hydrogen bonding or metal chelation. In fact, earlier works by Ikegami indicated that the static dipole of MS is not the main driving force of the J-aggregation and that intermolecular hydrogen bonding or metal chelation plays key roles for J-aggregation [11,12]. In other words, the J-band nature can be tuned at the air/water interface controlling the subphase conditions. In fact, the peak position of the J-band of the MS-containing films at the air/water interface changes in a relatively wide range of 590 to 620 nm depending on the subphase conditions, which indicates the existence of various polymorphs of the J-aggregate [1-12]. If various polymorphs of the MS J-aggregate can be transferred onto solid substrates controlling the subphase conditions, it is intriguing both from technological and scientific point of views.

It should be noted, however, that the J-bands tend to be transient at the air/water interface and the transfer of the floating monomolecular films with the target polymorph onto a solid substrate is often difficult [11-13]. Thus, in order to overcome the difficulty and realize LB films with various polymorphs of the MS J-aggregates, the application of secondary treatments to the dye LB film is effective.

The long-chain derivative of merocyanine (MS in Figure 1) is well known to form stable monolayers at the air/water interface when it is mixed with arachidic acid (C_20_ in Figure 1) [1-10].

The MS-C_20_ mixed monolayers formed on an aqueous subphase containing Cd^2+^ ions are easily transferred to solid substrates to form Langmuir-Blodgett (LB) films, which are blue in color in the as-deposited state due to the J-band with its peak located around 590 to 594 nm [2-5]. Thus, the MS-C_20_ binary LB system is suitable for applying secondary treatments to induce structural transitions. In fact, there are many reports on the color-phase transition of the MS-C_20_ binary LB system induced by various secondary treatments, such as acid treatments (ATs), basic treatments (BTs), and dry-heat treatments (DHTs) [5,7,14,15]. DHTs as well as ATs in both liquid and gas phases dissociate the J-band, with the film changing from blue to red [6,8]. On the other hand, BTs in both liquid and gas phases restore the J-band with the film color changing back to blue when applied to the red films after AT or DHT [5,7].

Recently, we have found that the hydrothermal treatment (HTT), which is a heat treatment under relative humidity of 100%, is effective for controlling the dye aggregation states when it is applied to the well-known MS-C_20_ binary LB film [16-26]. The as-deposited J-band originally located around 590 nm is reorganized by HTT to form a new phase associated with a further narrowing and a red shift of the peak [16-26].

We have already investigated kinetics of hydrothermally induced reorganization of J-aggregate in the mixed MS-C_20_ LB system and have pointed out that the UV-visible absorption spectra can be deconvoluted to three components: Band I (centered at 500 to 515 nm), Band II (centered at 545 to 555 nm), and Band III (centered at 590 to 598 nm) [17,19,22,26]. Band I, Band II, and Band III are assigned as the blue-shifted dimer, monomer, and red-shifted J-aggregate, respectively. Furthermore, the HTT process consists of following two stages. The first stage is characterized by the decrease in the Band III component associated with the increase in the Band I component, which is hypothesized as a dissociation process of the original J-aggregate (Band III centered at 590 nm) to the blue-shifted dimer (centered at 500 to 515 nm). The second stage is characterized as the reorganization of Band III (centered at 597 to 599 nm) from Band I (500 to 515 nm). Since the component of Band II (centered at 545 to 555 nm) is almost unchanged throughout the whole HTT process, we have described that the growth and decay processes in the second stage are assumed to be a first-order reaction between Band I and Band III components [22,26].

We have also reported that the HTT process induces a unique superstructure in the MS-C_20_ binary LB systems [18,20-25]. Giant round-shaped domains with diameters reaching 100 μm are observed by optical microscopy. In those papers, we have touched upon the sizes of the round-shaped domains depending on heating temperature (*T*_H_) and heating time (*t*_H_) and found that the average size of the domains tends to increase superlinearly depending on *T*_H_ and *t*_H_. However, due to insufficient color sensitivity and resolution of the optical microscope used for the observation, the surface structure had not been characterized in detail [18,20-25].

Since J-aggregate is known to emit intense fluorescence, fluorescence (FL) microscopy is considered to be a powerful tool to characterize the system. In this paper, we report on surface morphology of the MS-C_20_ binary LB films before and after HTT process combining bright field (BF) microscopy and FL microscopy and discuss the possible mechanisms of the J-aggregate reorganization.

## Methods

### Fabrication of the mixed LB films of Merocyanine and arachidic acid

The film-forming materials, merocyanine dye (MS in Figure 1) and arachidic acid (C_20_ in Figure 1), were purchased from Hayashibara Biochemical Lab. Inc. (Okayama, Japan) and Fluka AG (St. Gallen, Switzerland), respectively, and used without further purification. They were dissolved in optical-grade chloroform from Tokyo Kasei Kogyo (Tokyo, Japan) with a molar mixing ratio MS/C_20_ = 1:2. The monolayers were prepared on a Cd^2+^-containing subphase and successively deposited onto one side of a substrate using the conventional vertical dipping technique as described in our previous papers [18-21]. Glass substrates with a dimension of 38 × 13 × 1 mm^3^ cut from ordinary glass slide from Matsunami Glass Ind., Ltd. (Type S-1111, Kishiwada, Japan) were coated by five-layered LB films of cadmium arachidate prior to the deposition of the mixed MS-C_20_ LB film.

**Figure 2:**
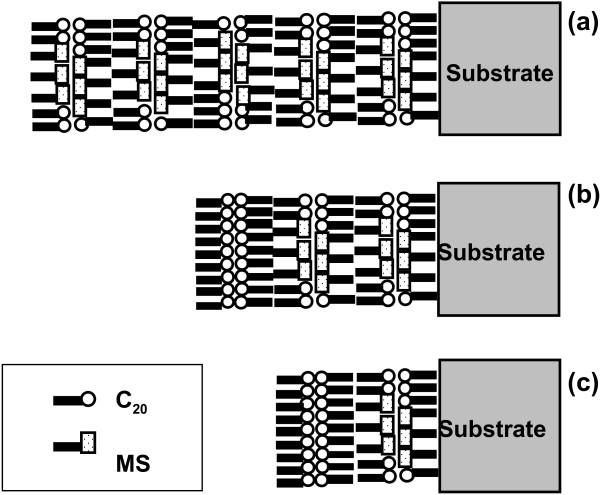
**Schematic representations of the layered structures of the as-deposited LB films of three different types. (a)** Ten layers of MS-C_20_ [10 × (MS-C_20_) /5 × (C_20_)/ glass], **(b)** four layers of MS-C_20_ [2 × (C_20_) /4 × (MS-C_20_) /5 × (C_20_) /glass], and **(c)** two layers of MS-C_20_ [2 × (C_20_)/4 × (MS-C_20_)/5 × (C_20_)/glass], where ‘2 × (C_20_)’ denotes a double layer of cadmium arachidate for coating the MS-C_20_ surface for stability.

Three different layered structures were constructed, as shown in Figure 2: in panel a, ten layers of MS-C_20_ [10 × (MS-C_20_)/5 × (C_20_)/glass]; in panel b, four layers of MS-C_20_ [2 × (C_20_)/4 × (MS-C_20_)/5 × (C_20_)/glass]; and in panel c, two layers of MS-C_20_ [2 × (C_20_)/4 × (MS-C_20_)/5 × (C_20_)/glass]. The surfaces of the four- and two-layered MS-C_20_ films were covered by double layers of cadmium arachidate [2 × (C_20_)] for stability, as shown in Figure 2a,b, respectively. The mixed MS-C_20_ LB systems were of Y-type with a deposition ratio of approximately unity for both upward and downward strokes.

### Hydrothermal treatment procedure

**Figure 3:**
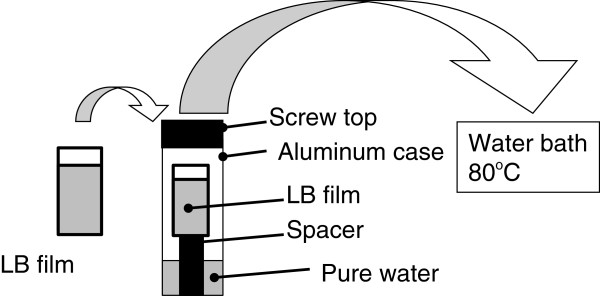
**The procedure of the hydrothermal treatment (HTT).** An aluminum tube (*ca.* 20 mm in diameter and 150 mm long) was first filled with pure water of 2 mL. The LB sample was put in the tube using a spacer to prevent direct contact of the sample with the water. Finally, the tube was closed by a screw lid using a Teflon tape and immersed in a water bath kept at 80°C.

The as-deposited films were put in an aluminum tube (*ca.* 20 mm in diameter and 150 mm long) together with pure water of 2 mL, as shown in Figure 3. The sample was raised to avoid direct immersion in water inside the aluminum tube using a spacer. After a screw lid was put on top of the case and tightly sealed using a Teflon tape, the case was immersed in a water bath kept at 80°C for 60 min and then cooled down to room temperature.^a^ In the aluminum tube of 50 mL, the amount of pure water (2 mL) is estimated to be enough to realize relative humidity of 100% with a positive pressure of about several bars during the heat treatment [21].

### Characterization of the mixed LB films of merocyanine and arachidic acid

The UV-visible absorbance spectra *A*_//_ and *A*_⟂_ were measured using a Shimadzu UV-2100 spectrophotometer (Kyoto, Japan), where *A*_//_ and *A*_⟂_ refer to linearly polarized incident lights with the electric vectors parallel and perpendicular to the dipping direction in the deposition processes, respectively. The BF microscopy and FL microscopy images were obtained using a Keyence BZ-8000 microscope (Osaka, Japan). Red fluorescent images were taken using a 540-nm excitation.

## Results and discussion

**Figure 4:**
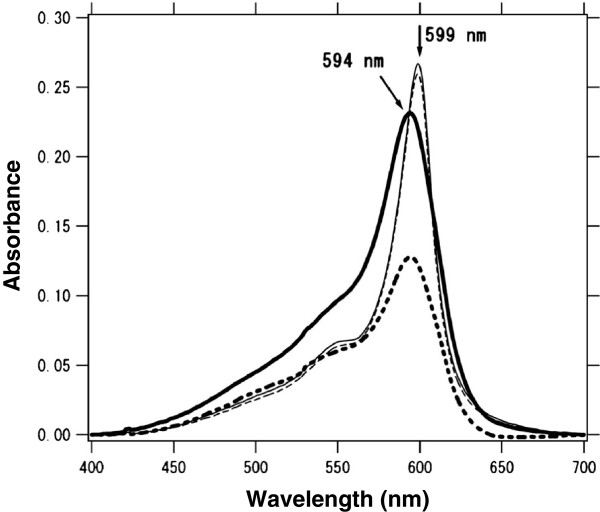
**Typical absorption spectra of a ten-layered MS-C**_**20 **_**binary LB film.** The thick solid and dashed lines represent *A*_//_ and *A*_⟂_of the as-deposited state, respectively; the thin solid and dashed lines represent *A*_//_ and *A*_⟂_ after hydrothermal treatment (HTT) at 80°C for 60 min.

Figure 4 shows typical UV-visible absorption spectra of the ten-layered LB film of MS and C_20_ with the molar mixing ratio of 1:2 before and after HTT (80°C, 60 min). The well-known J-band, which is located at 594 nm in the as-deposited state, shifts to 599 nm, as shown in Figure 4. The dichroic ratio *R* ≡ *A*_//_ / *A*_⟂_ = 1.81 at its peak around 594 nm before HTT but the anisotropy almost disappears (*R* = 1.03 at 599 nm) after HTT (80°C, 60 min), as shown in Figure 4. Furthermore, the band shape becomes appreciably sharper by HTT. These results are in good agreement with our previous works.

**Figure 5:**
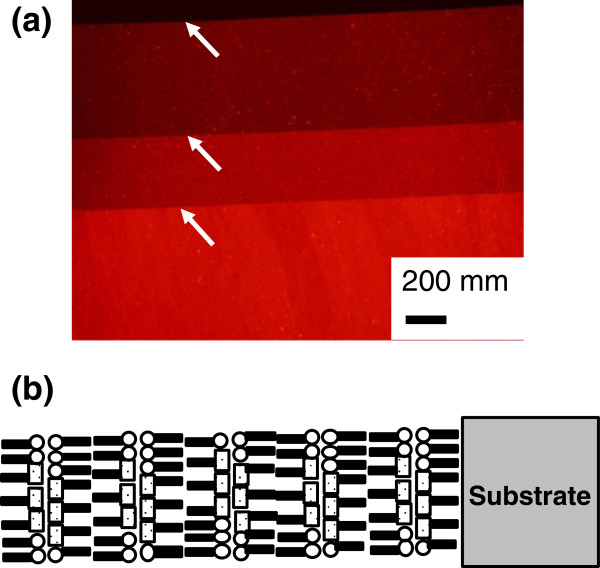
**A typical FL micrograph of the as-deposited MS-C**_**20 **_**binary LB film of ten layers.** Red fluorescent image with 540-nm excitation **(a)**; the schematic layered structure **(b)**.

Figure 5a shows a typical FL micrograph of the as-deposited MS-C_20_ LB film of ten layers with the schematic layered structure shown in Figure 5b. Intense red fluorescence is observed over the whole film area, and the intensity steps are clearly seen at monomolecular steps created by shifts of meniscus lines during the deposition process of the MS-C_20_ LB film, as shown by arrows in Figure 5a. It has been well known that MS and C_20_ are phase separated in MS-C_20_ binary LB system. Minari and coworkers estimated that the length of the MS J-aggregate as several hundred nanometers and that the MS J-aggregates are separated from the regions of matrix molecules of C_20_ based on the analytical model for characterizing the flow orientation effect during the transfer process of the LB deposition [27]. Kato and coworkers also indicated that the MS-C_20_ mixed system is phase separated into MS-rich (dye-rich) regions and C_20_-rich (fatty acid-rich) ones and that the MS-rich (dye-rich) regions are further separated into dye monomer regions and J-aggregate crystallites based on characterization by atomic force microscopy (AFM) observation, FL microscopy, and second harmonic generation (SHG) microscopy [9,28]. Kato and coworkers further estimate that the size of J-aggregate is in the range of 0.5 to 10 μm based on SHG microscopy observation. We hypothesize a similar mesoscopic texture in which the mixed ultrathin film is separated into MS-rich regions and C_20_-rich ones and the MS-rich regions are further separated into the dye monomer regions and J-aggregate crystallites in the as-deposited MS-C_20_ mixed system because the dye monomer band and J-band coexist at 545 to 555 nm and 594 nm, respectively, as shown in Figure 4. In fact, the surface of the as-deposited MS-C_20_ LB system observed by FL microscopy is almost featureless, as shown in Figure 5a, indicating that the crystallite sizes of J-aggregate are less than 10 μm.

**Figure 6:**
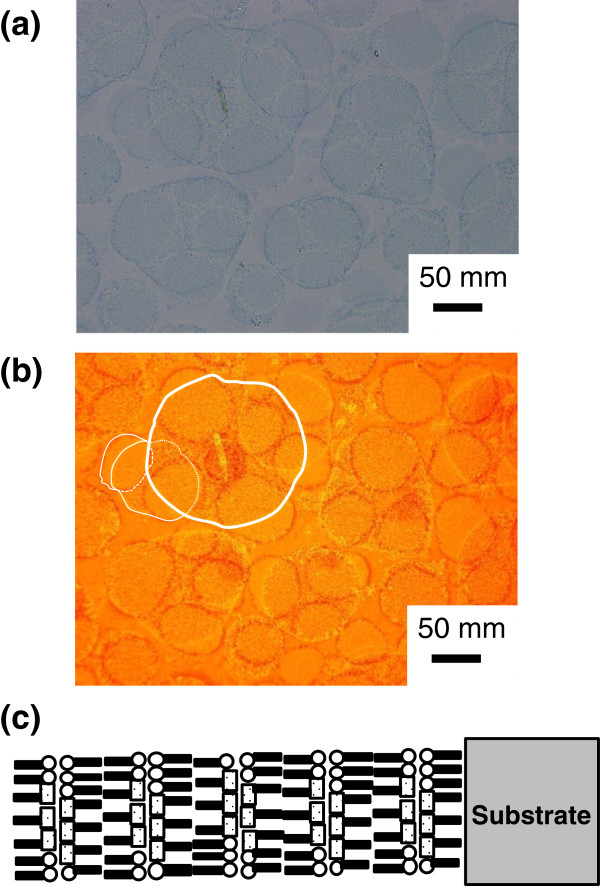
**A BF microscopy image and the FL microscopy image of the mixed MS-C**_**20 **_**LB film.** A BF microscopy image **(a)** and the FL microscopy image (red fluorescent image with 540-nm excitation) of the corresponding area **(b)** of the mixed MS-C_20_ LB film of ten layers after HTT (80°C, 60 min) with the schematic layered structure **(c)**.

Figure 6 shows the BF microscopy image (a) and the FL microscopy image (red fluorescent image with 540-nm excitation) of the MS-C_20_ mixed LB film of ten layers after HTT (80°C, 60 min) (b) together with the schematic layered structure (c). Round-shaped domains are observed both by BF microscopy and FL microscopy and the domain sizes are reaching 100 μm in diameter. In our previous works, due to insufficient color sensitivity and the resolution limit of the BF microscope, microstructures of the domains were not characterized sufficiently [18,20-25]. However, from Figure 6a in the present work, it has been found that the bluish areas tend to be observed in round-shaped domains compared to areas outside. Furthermore, the bluish areas observed by BF microscopy (Figure 6a) are found to emit intense fluorescence compared to colorless areas, as shown in Figure 6a,b. These results strongly indicate that the bluish areas emitting intense red fluorescence correspond to the crystallites of reorganized J-aggregates.

It should be also noted that there are two different types of domains observed in Figure 6a,b. One type is of domains with rims of deeper blue (blue-rimmed domains), and the other type is of domains with rims of lighter blue (white-rimmed domains). As shown in Figure 6b, the fluorescence image shows that the emission from blue rims is more intense compared to areas inside, and on the other hand, the emission from white rims is less intense compared to areas inside. Diameters of blue-rimmed domains are reaching 100 μm or even greater, as seen in Figure 6a,b. On the other hand, diameters of white-rimmed domains are typically in the range of 40 to 60 μm, which are significantly small compared to blue-rimmed domains.

In our previous works, we categorized the two types of domains as ‘dark-rimmed domains’ and ‘bright-rimmed domains’ [18,22], which are now categorized as blue-rimmed domains and white-rimmed domains, respectively. Observations by BF microscopy and FL microscopy have revealed that the crystallites of J-aggregates exist in domains of both types in the mixed MS-C_20_ LB films after HTT. Furthermore, in blue-rimmed domains, the density of reorganized J-aggregate crystallites appears to be higher near domain boundaries compared to other areas. On the other hand, in white-rimmed domains, the density of J-aggregate crystallites appears to be lower near domain boundaries compared to other areas. There is a certain tendency that white-rimmed domains occasionally stack on one another, while blue-rimmed domains are located above white-rimmed domains. This implies that white-rimmed domains are confined in the inner layers and blue-rimmed domains are located at the outermost monolayer, although the mechanism for the domain formation through HTT process is not clear at this stage.

As shown in Figure 6a,b, the domains tend to stack on one another, and a threefold stack is recognized, as shown by white schematic rims drawn in Figure 6b. Stacks up to three layers have been observed for many sample batches of the ten-layered mixed MS-C_20_ film, allowing us to estimate that the average thickness of the domains is less than four layers, which corresponds to *ca.* 10 nm.

**Figure 7:**
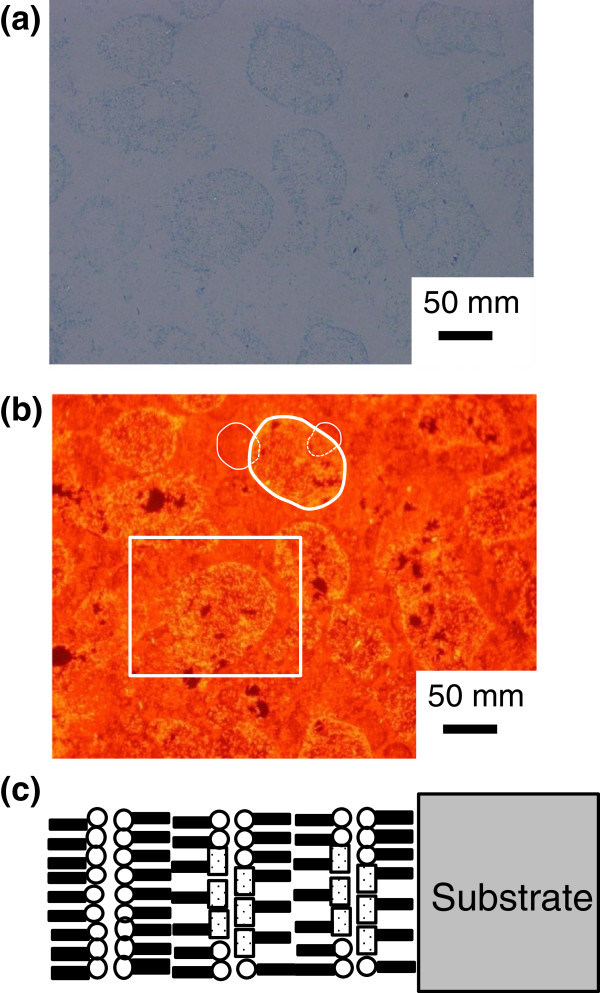
**A BF microscopy image and the FL microscopy image of the MS-C**_**20 **_**mixed LB film.** A BF microscopy image **(a)** and the FL microscopy image (red fluorescent image with 540-nm excitation) **(b)** of the MS-C_20_ mixed LB film of four layers after HTT (80°C, 60 min) with the schematic layered structure **(c)**. The surface of the MS-C_20_ binary LB film is covered by a double layer of cadmium arachidate.

Then, we reduced the number of layers in order to further investigate the microstructure and the thickness of the round-shape domains. Figure 7 shows a BF microscopy image (a) and the FL microscopy image (red fluorescent image with 540-nm excitation) (b) of the MS-C_20_ mixed LB film of four layers after HTT (80°C, 60 min) together with the schematic layered structure (c). As shown in Figure 7c, the outermost layer of the MS-C_20_ mixed LB film is covered by a double layer of cadmium arachidate (C_20_) for stability. Round-shaped domains are also observed by BF microscopy and FL microscopy. However, as seen in Figure 7a, rims of the domains are featureless compared to those observed in the ten-layered MS-C_20_ mixed LB systems. As shown by white schematic rims drawn in Figure 7b, a twofold stack is recognized. Thus, we further estimate that the average thickness of domains corresponds to a double layer or one single monolayer, i.e., <5 to 6 nm.

**Figure 8:**
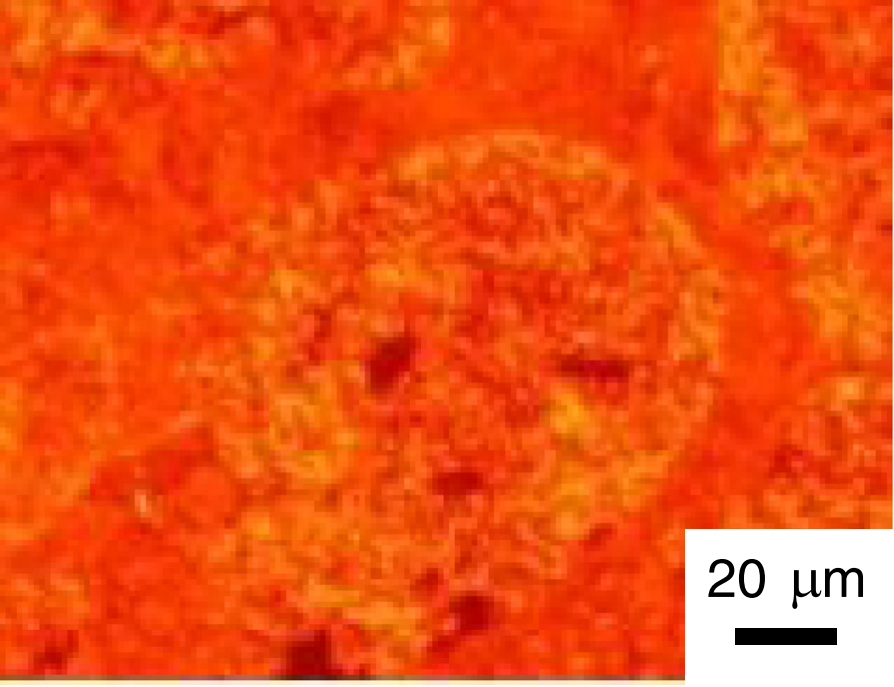
**Digitally magnified FL microscopy image within an area surrounded by the white frame drawn in Figure**7**b.**

Figure 8 shows a digitally magnified FL image within an area surrounded by the white frame drawn in Figure 7b. The round-shaped domains are filled with grains emitting intense fluorescence. It appears that the grain sizes are less than 10 μm. We postulate that those grains are of crystallites of J-aggregates reorganized by HTT process.

**Figure 9:**
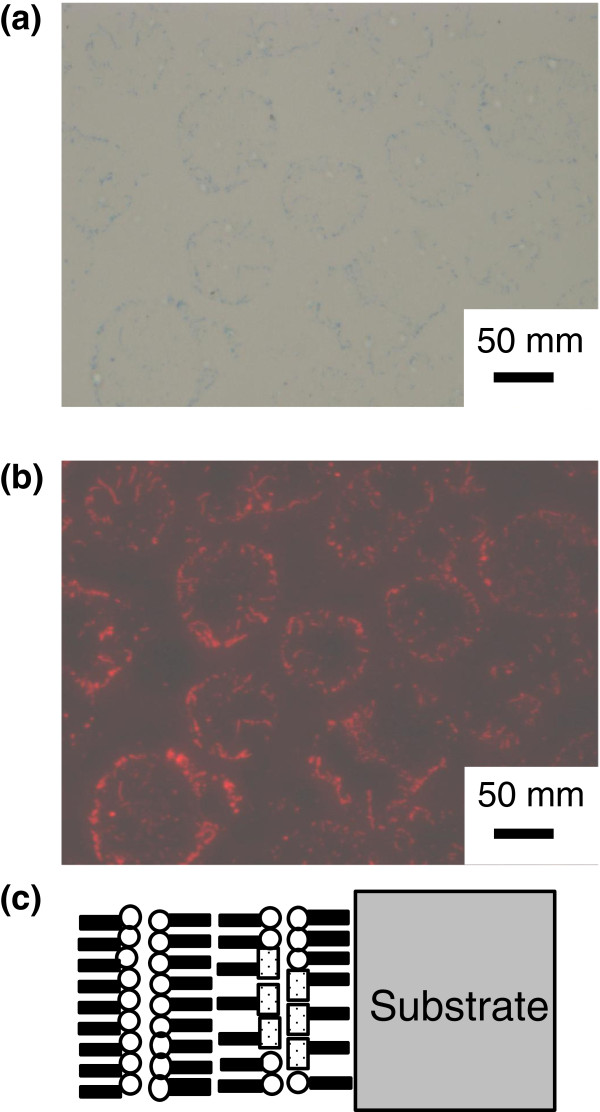
**A BF microscopy image and the FL microscopy image of the mixed MS-C**_**20 **_**LB film.** A BF microscopy image **(a)** and FL microscopy image (red fluorescent image with 540-nm excitation) **(b)** of the mixed MS-C_20_ LB film of two layers after HTT (80°C, 60 min) with the schematic layered structure **(c)**. The surface of the MS-C_20_ binary LB film is covered by a double layer of cadmium arachidate.

Finally, we further reduced the number of layers and investigated surface of the MS-C_20_ binary LB film. Figure 9 shows a BF microscopy image (a) and the FL microscopy image (red fluorescent image with 540-nm excitation) (b) of the MS-C_20_ mixed LB film of two layers after HTT (80°C, 60 min) together with the schematic layered structure (c). As shown in Figure 9c, the outermost layer of the MS-C_20_ mixed LB film is covered by a double layer of cadmium arachidate (C_20_) for stability. Round-shaped domains are also observed by BF microscopy and FL microscopy. As seen in Figure 9a, bluish areas tend to be located near domain boundaries in the two-layered MS-C_20_ mixed LB system. Furthermore, bluish areas near the boundaries observed by BF microscopy emit red fluorescence, as shown in Figure 9b. Stacks of domains are not observed. Thus, the estimated thickness of the domains, i.e., <5 to 6 nm, is considered to be reasonable.

We have already reported that the original J-band of the as-deposited MS-C_20_ binary LB systems (located at 590 to 594 nm) has a significant optical anisotropy due to the flow orientation effect during the transfer process [27], but the reorganized J-band located at 597 to 599 nm after HTT is isotropic, as shown in Figure 4. In our previous papers, we pointed out that the growth of the new phase of the J-band is well described by a first-order reaction between Band I (blue-shift-dimer band located at 500 to 515 nm) and Band III (J-band located in the range of 590 to 598 nm which includes both of the original band at 590 to 594 nm and the reorganized one at 597 to 599 nm), while the Band II component (monomer band located at 545 to 555) remains almost unchanged [17,19,22,26]. The reason of the optical isotropy of the reorganized J-band (at 597 to 599 nm) is considered to be due to that crystallites of the J-aggregate grow randomly in the film plane starting from the blue-shift dimers. This picture is in good agreement with the FL microscopy image in Figure 8, where we observe no significant tendency as for the growth direction of crystallites in the film plane. Therefore, it is reasonable to estimate that the reorganized J-band also has a certain optical anisotropy within each crystallite but it cancels each other by the random growth within the film plane.

**Figure 10:**
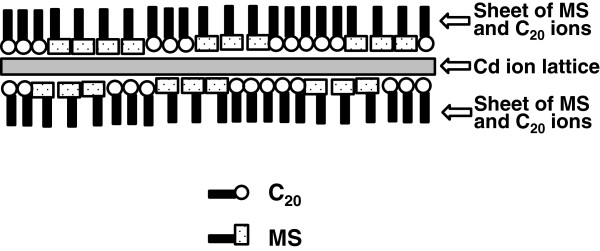
**A schematic representation of the bilayer unit cell of an MS-C**_**20 **_**binary LB film.**

Figure 10 shows a schematic representation of the bilayer unit cell of the MS-C_20_ mixed LB film. The bilayer unit cell can be described as a Cd^2+^ ion lattice sandwiched between a pair of negatively charged sheets, consisting of [C_20_]^−^ and [MS]^−^ anions with their CH_3_− and COO^−^ groups directed toward the outer and inner directions, respectively [16]. As the role of water, two different effects have been so far considered, i.e., the lubrication and hydration. The lubrication may reduce the energy barriers of microbrownian motions that are more or less hindered in the LB system, while the hydration effect may dissociate the ionic bonds, which stabilize the layered structure. The unit cell is stabilized by the ionic bonds between the positively charged Cd^2+^ ion lattice and the negatively charged pair of sheets and by the van der Waals force that gather those constituents. In the lubrication effect, the H_2_O molecules can reduce the van der Waals forces by their larger polarizabilities resulting in the reorganization of MS chromophores and the long hydrocarbon chains without significantly affecting the ionic bonds. On the other hand, the cell structure will be drastically changed by H^+^ and OH^−^ ions if they are incorporated in the film system, leading to degradation of the Cd^2+^ ion lattices in case the hydration effect predominates in the HTT process.

We have already reported the results on XRD analyses of the MS-C_20_ binary LB systems before and after the HTT processes [18,24]. The analyses revealed that the d-spacing of the as-deposited MS-C_20_ binary system is 5.52 nm, which corresponds to the well-known Cd-Cd spacing in the Y-type LB film of C_20_ (2 × 2.76 nm). By HTT, the positions of diffraction peaks remain almost unchanged, while the diffraction intensities remarkably increase associated with a narrowing in width. For instance, the intensity of the peak of fifth order increases by a factor of two by HTT. A similar change, i.e., the increase in peak intensity associated with the narrowing, is also observed when the dry-heat treatment (DHT, conventional annealing without water vapor) is applied to the same LB system. However, the J-band is not reorganized but simply dissociated by heat treatment without water molecules (DHT). Therefore, we consider that the lubrication effect by the presence of water molecules predominates in the HTT process.

In order to further investigate the surface structure of the dye-fatty acid mixed system, topographic characterization by atomic force microscopy is also worth performing and these will be reported elsewhere.

## Conclusions

We have characterized the mixed LB films based on merocyanine dye (MS) and arachidic acid (C_20_) focusing on the morphology studied by BF microscopy and FL microscopy. The results are summarized: (1) the as-deposited MS-C_20_ mixed LB film with molar mixing ratio MS/C_20_ = 1:2 emit intense red fluorescence uniformly over the whole film area by 540-nm excitation indicating that MS and C_20_ are phase-separated and the crystallite sizes of the J-aggregate are less than 10 μm, (2) by hydrothermal treatment (HTT), round-shaped domains, whose sizes are reaching 100 μm in diameter, emerge in the LB systems, (3) crystallites of J-aggregates tend to be in the round-shaped domains compared to the outside area in the film, (4) there are two different types of domains, i.e., blue-rimmed domains and white-rimmed domains, which are postulated to be confined in inner layers and at the outermost layer, respectively, and (5) the thickness of the domains is equal to or less than that of the double layer of the MS-C_20_ mixed LB film, which is *ca.* 5.52 nm.

The molecular order of MS in the J-aggregate is improved by the HTT process leading to the significant sharpening of the band shape together with the further red shift of the band (from 590 nm up to 597 to 599 nm). However, owing to the random growth of the J-aggregate in the film plane, the reorganized J-band is ‘apparently’ isotropic. As the role of water, two different effects have been so far considered, i.e., the lubrication and hydration. We consider that the lubrication effect by the presence of water molecules contributes dominantly to the reorganization of J-aggregate while the hydration contributes a small or even negative part in the HTT process.

## Endnotes

^a^We have already reported that the hydrothermal treatment (HTT) in the temperature range of 30°C to 90°C can reorganize the original J-band to form the new J-band phase located at around 600 nm. We set the temperature of HTT at 80°C because the average diameter of the round domains is largest after HTT at 80°C in the temperature range of 30°C to 90°C [21].

## Competing interests

The authors declare that they have no competing interests.

## Authors’ contributions

YFM prepared the Langmuir-Blodgett films and characterized the morphology by bright field microscopy and fluorescence microscopy. The spectroscopy characterization has been performed by YFM, MS, and TS. YFM directed the research and prepared the draft of the manuscript. The manuscript has been further revised based on discussions among YFM, MS, and TS. All authors read and approved the final manuscript.
